# Birth Defects Related to Prenatal Exposure to Essential and Toxic Elements

**DOI:** 10.7759/cureus.113265

**Published:** 2026-07-23

**Authors:** Jesse Cottrell, Kimberly Sullivan, Danielle Frieson, Wondwosen Yimer, Bianca Key, Babbette LaMarca

**Affiliations:** 1 Department of Obstetrics and Gynecology, Marshall University Joan C. Edwards School of Medicine, Huntington, USA; 2 Department of Obstetrics and Gynecology, University of Mississippi Medical Center, Jackson, USA; 3 Department of Pharmacology, University of Mississippi Medical Center, Jackson, USA

**Keywords:** birth defects, elements, fetus, pregnancy, prenatal, toxic, toxicology

## Abstract

Background

All naturally occurring metals and other elements are present in at least trace concentrations in soil, water, air, and organisms. Accumulation of these elements can lead to developmental delays, neurological damage, and even death. Although the toxicities associated with metals such as lead or mercury are well-demonstrated, little data exists regarding birth defects and maternal environmental exposures to other trace essential or toxic elements.

Methodology

A prospective case-control study of 23 patients delivering at the University of Mississippi Medical Center with prenatally diagnosed birth defects (N = 18) was performed. Control participants (N = 5) were selected for comparison. Maternal and fetal serum concentrations of essential and toxic elements were measured at delivery and analyzed for associations with fetal birth defects, geographic location, patient demographics, and perinatal outcomes.

Results

Copper and zinc levels were higher in maternal groups compared to fetal levels. Manganese levels were higher in fetal samples. We were unable to demonstrate any associations between birth defects and toxic element levels. On secondary analysis, higher levels of copper were found in the maternal serum of mothers who lived in an urban location (p = 0.03), while magnesium levels were found to be higher in mothers in rural locations (p < 0.01).

Conclusions

Although there was a significant difference between levels of copper and magnesium based on the geographic locations of mothers and offspring, there was no direct correspondence between maternal and neonatal element levels and the development of birth defects in our study.

## Introduction

All naturally occurring metals and other elements are present in at least trace concentrations in soil, water, air, and organisms. Under certain conditions, these elements can accumulate to high concentrations, leading to developmental delays, neurological damage, and even death [1-5]. Due to the long-term consequences of prenatal exposure, many reproductive health professional societies have advocated for further research and awareness regarding prenatal exposure to essential and toxic elements [4].

Adverse fetal outcomes are most often associated with toxicity due to heavy metals such as lead or mercury [6-8], and little data exists to clearly establish a link between birth defects and maternal environmental exposures to trace essential elements or toxic elements. The importance of fetal birth defects on healthcare cannot be understated. The Centers for Disease Control and Prevention reports that major birth defects affect approximately 3% of births and contribute greatly to both infant mortality and billions of dollars in healthcare costs [9].

The underlying cause of most birth defects is unknown, with many genetic and environmental interactions theorized. The proposed mechanisms range from micronutrient deficiencies to DNA alteration through epigenetic changes. Prior studies have shown differences between urban and rural samples, and maternal serum levels of essential and toxic elements are thought to be influenced by diet, water source, and occupation [10].

The University of Mississippi Medical Center is a tertiary referral center for congenital birth defects. Given the varying environmental exposures of our rural and urban populations, we sought to determine whether there is a causal link between birth defects and prenatal exposure to excess or inadequate essential and toxic elements. Secondarily, we sought to determine if demographic differences exist based on maternal factors.

This data was presented at the American College of Obstetrics and Gynecology Annual Clinical and Scientific Meeting, April 30 to May 2, 2021, as a Virtual Presentation.

## Materials and methods

A prospective case-control study involving 23 patients was conducted to measure maternal and fetal serum concentrations of essential and toxic elements. The study was approved by the Institutional Review Board at the University of Mississippi Medical Center (approval number: 2016-0328). The collection of samples occurred between December 12, 2018, and April 3, 2019. Patients delivering at the University of Mississippi Medical Center in Jackson, MS, with prenatally diagnosed birth defects (N = 18) were recruited and consented in writing before delivery. Control participants with no prenatally diagnosed birth defects (N = 5) who underwent fetal anatomic survey at our institution were selected, recruited, and consented in writing for comparison. These controls were screened and consented while on labor and delivery when presenting for delivery after it was confirmed that a normal fetal anatomic survey was performed at 18-22 weeks of gestation. All study participants had fetal anatomic surveys performed via ultrasound by maternal-fetal medicine specialists and delivered at the University of Mississippi Medical Center, Jackson, MS.

Inclusion criteria for all patients included term gestation and collection of maternal venous blood and fetal umbilical cord blood. Exclusion criteria were non-collection of cord blood or inadequate collection. Inclusion criteria for controls included term gestational age with a completed anatomy scan. Exclusion criteria included known chromosomal abnormalities (birth defect or BD group), multi-fetal gestation, pre-existing chronic medical condition, preterm gestational age (control), inadequate prenatal care (no anatomy scan), and intravenous magnesium infusion.

Demographic information was extracted from each patient’s electronic medical record. Maternal and fetal serum concentrations of essential and toxic elements were measured at delivery and analyzed for associations with fetal birth defects, geographic location, patient demographics, and perinatal outcomes.

Essential elements that were measured included calcium, copper, lithium, magnesium, manganese, molybdenum, selenium, strontium, and zinc. Toxic elements that were measured included arsenic, barium, cadmium, cobalt, lead, mercury, nickel, platinum, thallium, and uranium. Umbilical cord blood was analyzed for 20 inorganic elements by The Great Plains Laboratory, Inc., located in Lenexa, KS. According to the lab protocol, The Great Plains Laboratory analyzes the elements in whole blood by inductively coupled plasma mass spectroscopy following specimen digestion with nitric acid in a closed-containing microwave oven system. The procedure measures the total concentration of an element in whole blood, regardless of biochemical form or partitioning of the element in blood fractions.

Comparisons and p-values between element levels and development of fetal birth defects were evaluated using the Mann-Whitney U test using R Software, version 3.3.1 (R Foundation for Statistical Computing, Vienna, Austria). Secondarily, baseline values of the essential and toxic elements for gravid mothers in the metro Jackson, MS, area and surrounding areas were compared between the groups.

## Results

In total, 23 maternal-fetal pairs were enrolled: 18 in the birth defect group and five in the control group. Birth defects included cardiac and brain abnormalities. There were no significant differences in the demographic variables of age, body mass index, gravidity, parity, or gestational age between the group with a known birth defect and the controls (Table 1).

**Table 1:** Demographics of the study population. BMI = body mass index; SD = standard deviation

Birth defect	n (%)	Variable	Mean	SD	Minimum	Maximum
No	5 (22)	Age	27.4	6.1	23	37
		BMI	31.2	4.6	25	37
		Gravida	3.8	1.3	3	6
		Parity	2.6	0.9	2	4
		Gestational age	38.2	1.1	37	39.2
Yes	18 (78)	Age	26.9	4.4	21	39
		BMI	31.9	7.3	20	47
		Gravida	3.1	1.3	1	6
		Parity	1.6	1.0	0	4
		Gestational age	37.4	2.3	30	39.2

The birth defect group was more likely to have private, VA, or other insurance listed, but the majority of both groups had Medicaid/Medicare as their payer source (Table 2).

**Table 2:** Insurance type.

Insurance type	Birth defect		
	No	Yes	Total
Private	1	4	5
Medicaid/Medicare	4	11	15
Veteran affairs	0	2	2
Other	0	1	1
Total	5	18	23

Regarding race, 3 (60%) patients in the control group and 11 (61%) patients in the birth defect group were Black (Table 3).

**Table 3:** Race of the study population.

Race	Birth defect		
	No	Yes	Total
Black	3	11	14
White	1	7	8
Other	1	0	1
Total	5	18	23

**Figure 1:**
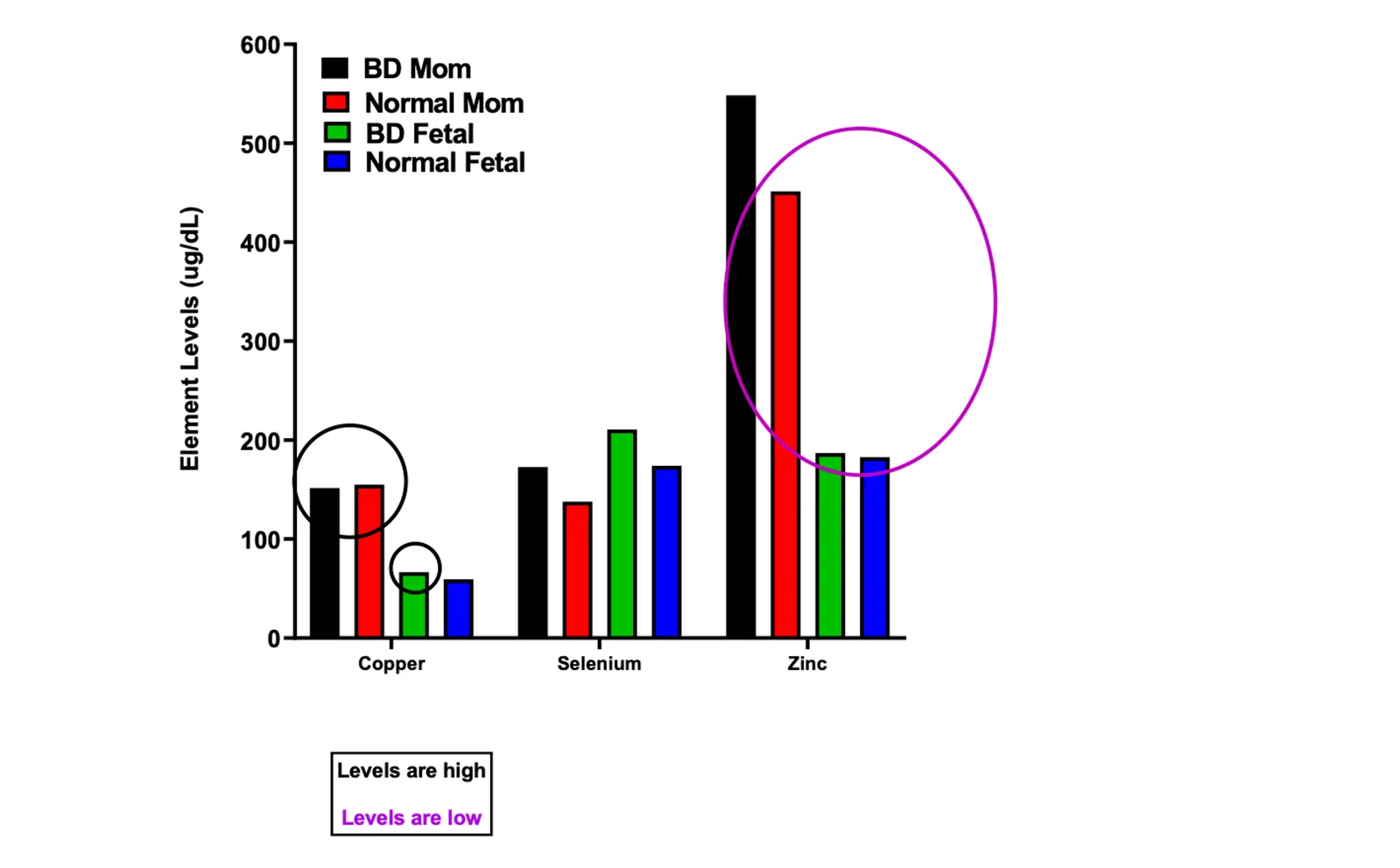
Copper, selenium, and zinc levels. BD = birth defect

For each of the essential elements we reviewed, we calculated both maternal and fetal levels (via cord blood). From this data, copper and zinc levels were higher in both maternal groups versus fetal levels (Figure 1). Copper levels were also found to be lower than expected, and zinc levels were higher than expected.

**Figure 2:**
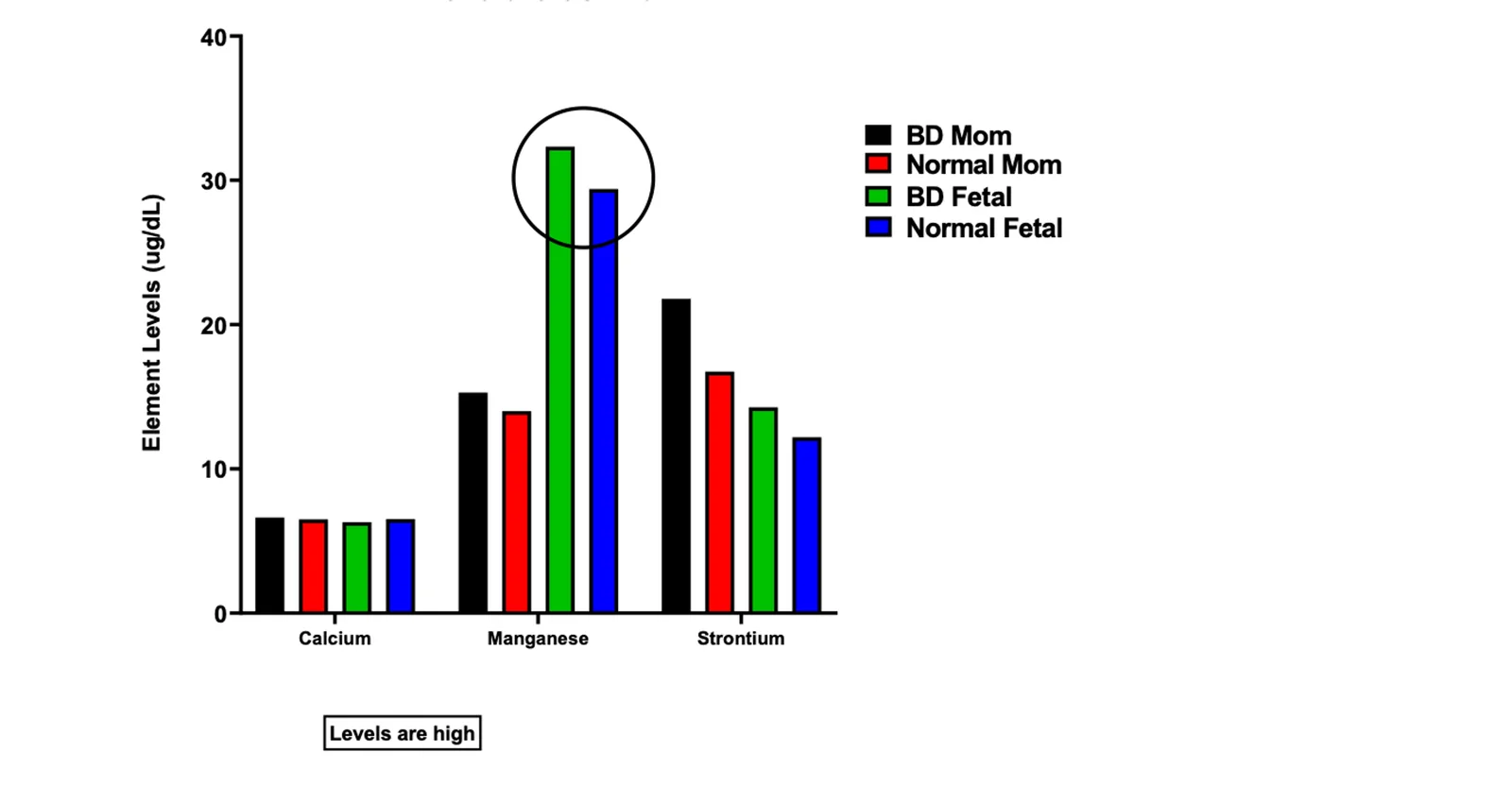
Calcium, manganese, and strontium levels. BD = birth defect

Manganese levels were higher in fetal samples compared to non-anomalous infants and those with birth defects (Figure 2).

**Figure 3:**
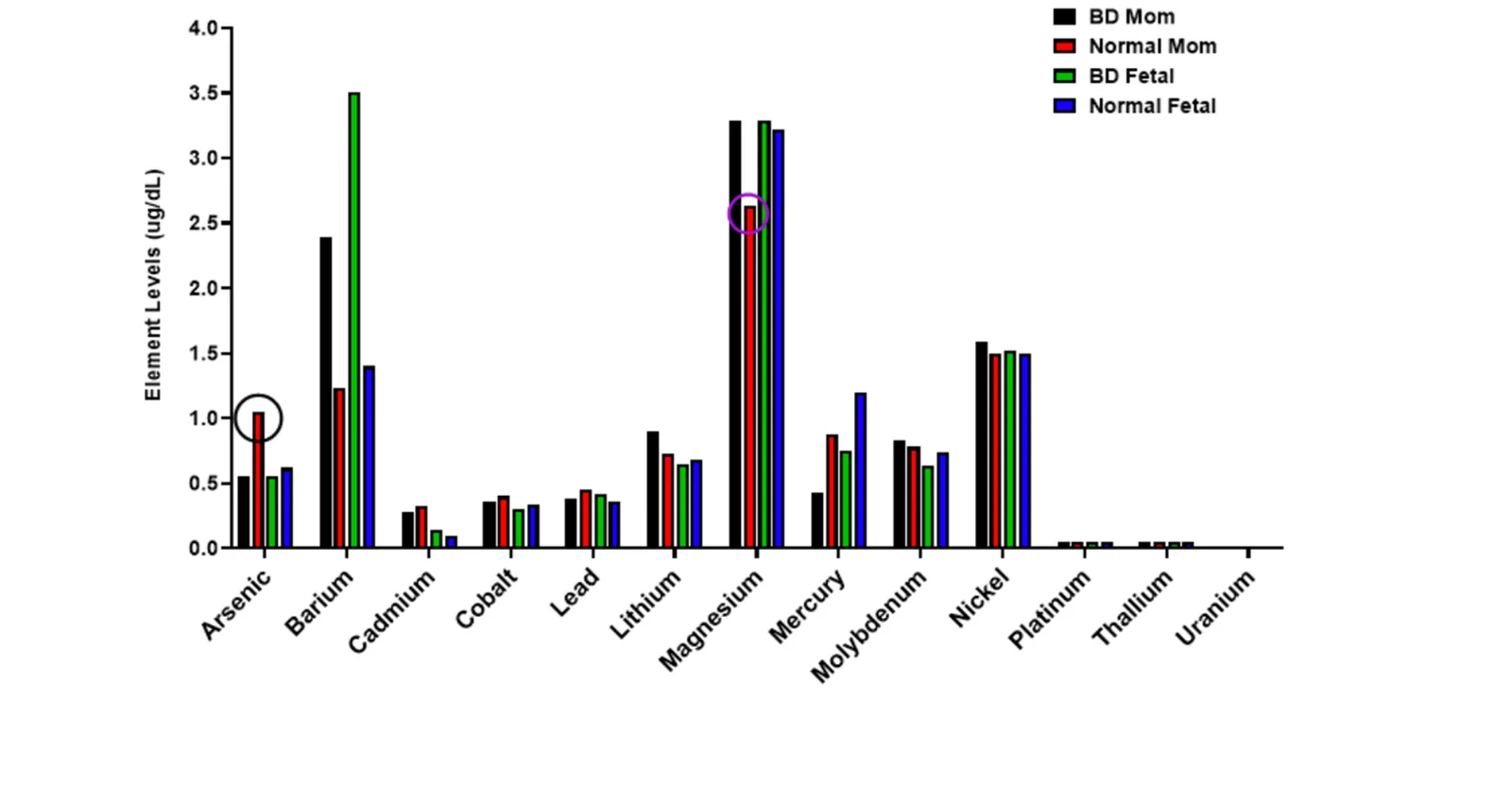
Toxic element levels. BD = birth defect

No associations between birth defects and toxic element levels were demonstrated in the study (Figure 3).

Overall, 80% of the controls were mothers who lived in an urban location, while 61% of the birth defect group lived in rural areas (Table 4). This finding was expected as most patients were referrals for tertiary care.

**Table 4:** Urban versus rural. BD = birth defect

Fetal BD	Urban, n (%)	Rural, n (%)	P-value
Yes	7 (38.9)	11 (61.1)	1.0
No	4 (80)	1 (20)	

On secondary analysis, there was a significant difference in levels of copper and magnesium based on geographic location. Higher levels of copper were found in the maternal serum of mothers who lived in an urban location, while magnesium levels were found to be higher in mothers living in rural locations (Table 5).

**Table 5:** Mean metal levels based on urban versus rural location.

Metal	Urban	Rural	P-value
Arsenic	0.8	0.5	0.22
Barium	1.31	3.15	0.42
Cadmium	0.31	0.26	0.36
Calcium	6.46	6.78	0.28
Cobalt	0.35	0.39	0.36
Copper	163.5	138.3	0.03
Lead	0.45	0.33	0.10
Lithium	0.58	1.21	0.28
Magnesium	2.72	3.66	<0.01
Manganese	15.4	14.5	0.80
Mercury	0.66	0.36	0.16
Molybdenum	0.9	0.71	0.42
Nickel	1.5	1.66	0.44
Platinum	0.05	0.05	1.0
Selenium	163.7	166.5	0.71
Strontium	19.3	22.38	0.84
Thallium	0.05	0.02	1.0
Uranium	0.02	0.02	1.0
Zinc	497.4	563.6	0.21

## Discussion

In our study, there was no direct association between maternal and fetal element levels and fetal birth defects when evaluated collectively. Copper and magnesium levels were significantly different (p < 0.03 and p < 0.01, respectively) based on the geographic locations of mothers and offspring in the Jackson, MS, area.

Fetal birth defects affect approximately 3% of all pregnancies, accounting for 20% of all infant deaths [9]. The suspected factors causing these birth defects range from genetic to environmental causes. Many prior studies have examined the relationship between essential and toxic elements and long-term neurodevelopmental outcomes; however, studies involving environmental exposure and structural birth defects are less robust. Zeyrek et al. aimed to evaluate the relationship between serum micronutrient concentration and occurrence of neural tube defects and found a significantly higher mean copper concentration in the maternal and umbilical cord blood samples of their study participants with known neural tube defects [11]. Elevated concentrations of molybdenum may also be associated with a reduced risk for orofacial clefts [12]. Increased cobalt levels are associated with a decreased risk for neural tube defects, as well as decreased risk with increased levels of the six essential trace elements, suggesting a protective effect [13]. Numerous animal studies have shown teratogenicity with maternal cadmium exposure and orofacial clefts in the offspring [10,14,15]. Environmental exposure can also lead to DNA damage, with Wang et al. demonstrating a correlation between folate deficiency and DNA damage in chromium workers [15].

Heart defects are one of the most common congenital birth defects, and a recent meta-analysis found significant associations between arsenic, cadmium, mercury, and lead exposure during pregnancy and an increased risk of congenital heart defects [10]. The analysis included 13 studies, and pooled odds ratios for arsenic, cadmium, mercury, and lead were 2.12, 1.30, 1.22, and 2.30, respectively, for total congenital heart defects.

Importantly, heavy metals have been shown to not only cause birth defects but also contribute to other adverse pregnancy outcomes. Consistent with prior studies, Bommarito et al concluded that trace urinary metals may disrupt placentation and be associated with preeclampsia [2]. Likewise, lead and cadmium exposure is associated with a higher incidence of preterm birth [9].

Regarding geographic incidence in the Jackson, MS metropolitan area, there are no prior studies examining birth defects and environmental factors. A prior study at our institution did demonstrate an increased incidence of the fetal birth defect gastroschisis between 2000 and 2008, but a suspected cause for this increase was not hypothesized by the authors [16]. Overwhelmingly, the evidence indicates a need for further research into birth defects and adverse pregnancy outcomes associated with prenatal exposure to essential and toxic elements. While some of these factors are non-modifiable, it is thought that environmental exposure may serve as a point of intervention in the prevention of both maternal and neonatal morbidity and mortality [17].

This study is the first in our geographic area to evaluate trace and toxic element exposure on the development of fetal birth defects. The study was strengthened by the prospective nature of the study, as prenatal birth defects were identified in utero. The limitations of the study include its small sample size and the wide variety of birth defects that we evaluated due to an overall low prevalence of fetal malformations. Another limitation is the collection of maternal and neonatal blood samples after the period of organogenesis. In future studies, the measurement of these essential and toxic elements would ideally be analyzed during the period of organogenesis.

## Conclusions

Our results do not indicate an association between maternal and fetal element levels and fetal birth defects when evaluated collectively. Analysis of a larger volume of study participants grouped by type of birth defect is recommended in future studies.
